# Information needs of informal caregivers in caring and rehabilitation for dioxin victims in Vietnam

**DOI:** 10.1186/s12889-023-15095-y

**Published:** 2023-07-24

**Authors:** Hien Thi Ho, Chi Linh Bui, Olinda Santin, Huong Thi Nguyen, Hien Luong Thi Nguyen, Hung Chi Do, Nghi Ngoc Tran, Hanh Thi Tuyet Tran, Anh Mai Nguyen, Gillian Carter, Ha Thi Thu Bui, Minh Van Hoang

**Affiliations:** 1 Faculty of Health and Medical Sciences, the University of Adelaide, Adelaide, South Australia Australia; 2 School of Nursing & Midwifery, Faculty of Medicine, Health & Life Sciences, Queen’s University Belfast, 97 Lisburn Road, Belfast, UK; 3 Faculty of Clinical Medicine, Hanoi University of Public Health, 1A Duc Thang, Hanoi, Vietnam; 4 E Central Hospital, 89 Tran Cung st, Hanoi, Vietnam; 5 Medical Services Administration Department, Ministry of Health, Hanoi, Vietnam; 6 Hanoi University of Public Health, Hanoi, Vietnam

**Keywords:** Caregivers, Care, Information needs, Carers, Rehabilitation, Dioxin, Agent Orange, Vietnam

## Abstract

**Background:**

Health outcomes among Agent Orange/dioxin (dioxin) victims are significant due to many individuals requiring daily assistance, informal care, and rehabilitation support. This study aimed to identify the information needs of informal caregivers of dioxin victims in Vietnam.

**Methods:**

A cross-sectional study was conducted in Quynh Phu district, Thai Binh province – an area with a large number of dioxin victims, from June 2019 to June 2020. Quantitative data were collected from 124 caregivers of victims via structured interviews. Qualitative data were collected using semi-structured interview guides with in-depth interviews (IDI) (n = 36) and two focus group discussions (FGD) (n = 12).

**Results:**

The results demonstrated that all caregivers of dioxin victims were family members, predominantly older (71.8%), 61.5 years old on average, living on low incomes (87.9%), and were farmers (80.7%). Almost all participants (96.8%) reported having information needs, particularly concerning dioxin’s harms, nutrition, dioxin-related policies and rehabilitation, and psychological support for patients. Caregivers reported that they would like to receive information via health staff counselling (85.0%), television (75.0%), and community loudspeaker (65.8%). Notably, the majority of caregivers reported the need for information regarding psychological support (70.0%). These findings are consistent with qualitative data, which identify an urgent need to provide information, especially through health staff and digital resources.

**Conclusion:**

Many families with dioxin victims lived with little support and information, highlighting their high demand for information about care and rehabilitation. Thus, the healthcare system should promote information support, policy, and psychological support for caregivers and victims. An online support system for caregivers and victims is also recommended.

## Introduction

From 1961 to 1972, 74,175,920 liters of herbicides, of which Agent Orange (AO) accounted for 43,332,640 L, were sprayed over South Vietnam during the Vietnam War [1]. The term AO/dioxin and dioxin-like compounds (herein after called dioxin) include 75 individual compounds of polychlorodibenzo-p-dioxins (PCDD), 135 PCDF compounds (polychlorodibenzo-furans), and 209 PCB individual compounds (polychlorobiphenyls) [2]. The most toxic compound of the dioxin family is 2,3,7,8-tetrachlorodibenzo-p-dioxin (TCDD), which was a by-product presented in dioxin and other defoliants that the United States army used during the Vietnam War. Dioxin is classified into Group I carcinogens [3] and in addition to cancer, exposure to dioxin has been linked to adverse physical, developmental, reproductive, and other health issues [4]. Furthermore, research studies show that dioxin exposure is associated with health conditions in offspring of dioxin victims, such as preterm birth, intrauterine growth retardation, developmental delays, and other birth defects [5–7]. A recent longitudinal study, for example, shows that children living in a dioxin-contaminated area have a higher risk of low cognitive, language, and motor functions [7]. During the Vietnam War, approximately 4.8 million individuals were exposed to dioxin and other defoliants, illustrating the extensive impact and health concerns for Vietnamese citizens [6–10].

Health impacts among dioxin victims are significant with many individuals requiring daily assistance, informal care, and rehabilitation support. The Vietnam Ministry of Health recognized 17 diseases associated with dioxin exposure, such as cancer, spina bifida, and mental disorders, which cause functional impairments, activity, and participation restrictions. Victims need long-term care and rehabilitation at home and in the community. Long-term care and rehabilitation consist of many aspects, such as daily activity support, psychological support, assistive technology, and home-rehabilitation therapies [11]. People who are particularly vulnerable to dioxin, for instance, individuals who participated in the Vietnam War and residents who are living in surrounding hotspots, need to be examined for associated health conditions, as recommended by the Ministry of Health and Ministry of Labor, Invalids and Social Affairs. There is also an inter-ministerial circular on the guidance of examination for verification of diseases related to dioxin among people participating in the Vietnam War by the Ministry of Health and Ministry of Labor, Invalids and Social Affairs [12].

Despite the recognition of the severe effects of dioxin and the extensive medical and psychosocial support required, many families of victims have been living with limited assistance and support. The current health system in Vietnam, like many other low-and-middle-income countries (LMICs), is under-resourced and overcrowded. Healthcare support targets people requiring acute care, such as individuals receiving inpatient treatments, and little support is available for people living with illnesses in the community. Due to the lack of healthcare resources in Vietnam, the burden of support and care falls upon informal caregivers who are usually family members [13]. Despite managing a crucial role in the care and support of patients, informal caregivers in Vietnam often lack information and knowledge about caring and rehabilitation for patients. This lack of information and support results in caregivers feeling ill-equipped to manage the role [14]. Moreover, social policies and medical services for these caregivers have been limited nationally [15]. The 1,417,000 VN dong (equivalent to 61 US dollars) monthly support is only for the primary family caregiver of a dioxin victim with over 81% labour capacity decrease. These supports have been minimal and not enough to meet the basic needs of victims and their caregivers [16]. In addition, there is very limited empirical evidence regarding how we can best support families of dioxin victims.

It is anticipated that providing lifelong care leads to a heavy burden on caregivers of dioxin victims due to daily care and rehabilitation, financial support, emotional support, and support to access to health and social services. Evidence from other chronic health conditions, such as cancer, dementia, and children with disability highlights the reciprocal association between the health conditions and quality of caring tasks and the quality of life of caregivers [17–20]. Informal caregivers of chronic conditions face challenges because of lacking precise information; they need information about treatment cost, treatment methods, the prognosis of diseases, care package, and especially psychological support [21]. Understanding their needs is vital to the development of a supportive program to address caregivers’ problems, such as their lack of expertise and efficacy for the improvement of the quality of care and rehabilitation for patients [22, 23].

The limited evidence about the health and needs of caregivers in Vietnam mainly focuses on caregivers of dementia and cancer patients [14]. To our knowledge, there is no scientific data regarding the needs of caregivers of dioxin victims, especially regarding the information needs of this target group. Therefore, the study aimed to profile caregivers of dioxin victims and understand their information needs in caring and rehabilitation for these victims. This paper reports the results of caregivers’ information needs in Quynh Phu district, Thai Binh province, Vietnam, a distinct area with a large number of dioxin victims in Vietnam.

## Materials and methods

This study utilized a cross-sectional research design. Ethical approval was granted by the ethical committee in Vietnam before the implementation of the study. Dioxin caregivers were invited to participate if they met the following criteria: 18 years old or over, identified as the main caregivers of dioxin victims, have been providing care for dioxin victims for at least one year. The sampling of participants for the quantitative component was conducted in three stages. At the first stage, eight communes were randomly selected from the list of 37 communes in Quynh Phu district. At the second stage, 124 dioxin victims were recruited randomly from selected communes. The list of dioxin victims was taken from victim management records of commune health stations of Quynh Phu district. At the third stage, community health workers contacted them via telephone to invite the dioxin caregivers and other participants to participate in the study. As there is no validated instrument developed to measure the needs of dioxin caregivers, a structured questionnaire was developed. This questionnaire was based on scientific information about the information needs of caregivers of chronic health conditions [24] and the content of care and community-based rehabilitation in Vietnam. Specifically, the study used guidelines for community-based rehabilitation activities issued by the Vietnam Ministry of Health. These guidelines emphasized specific information on caring tasks, necessary knowledge, and skills of dioxin caregivers [25].

For the qualitative component (n = 48), purposive sampling was used to recruit individuals to participate in either two focus group discussions (FGD) or in-depth interviews IDI (36) with dioxin caregivers (n = 18), dioxin victims (n = 10), and community-based rehabilitation staff of communes and Quynh Phu district (n = 8).

The data was collected from June 2019 to June 2020. Quantitative data were entered using EpiData 3.1 software, cleaned, and analyzed using SPSS software version 18.0. Descriptive analysis was conducted. Qualitative data were transcribed verbatim, and thematic analysis was used. Coding reliability was the type of thematic analysis in the study, which is an inductive approach to qualitative analysis [26]. The approach was thoroughly described and guided by Braun and Clarke [26, 27]. The process of coding was conducted by four independent coders, and the level of agreement between the coders ranged from 0.89 to 0.95 (Cohen’s kappa).

## Results

The findings highlighted that the majority of caregivers of dioxin victims are wives and parents, and almost all participants have a high need for information about the caring and rehabilitation for victims.

### Characteristics of dioxin caregivers

In total, 124 dioxin caregivers participated in the study, of which 71/124 (57.3%) participants were women. The average age (min-max) of caregivers was 61.5 (32–79) years old, 71.8% were elderly people. Most of them graduated from secondary school (61.3%) and lived in rural areas (96.8%). The majority (87.9%) had a monthly low income of 1,000,000-1500,000 VND - equivalent to $43–65 USD, and the majority of participants were farmers (80.7%) (Table 1).

**Table 1:** Characteristics of dioxin victims’ caregivers

Characteristics		n (%)
Gender	Male	53 (42.7%)
	Female	71 (57.3%)
Mean age (years old)		61.5 (32–79)
Age groups (years old)	18–44	18 (14.5%)
	45–59	17 (13.7%)
	≥ 60	89 (71.8%)
Education levels	Primary school	9 (7.3%)
	Secondary school	76 (61.3%)
	High school	18 (14.5%)
	College	11 (8.9%)
	University	8 (6.4%)
	Others	2 (1.6%)
Location	Rural area	120 (96.8%)
	Urban area	4 (3.2%)
Monthly income (VND)	700.000–1.000.000	13 (10.5%)
	1.000.000-1.500.000	109 (87.9%)
	> 1.500.000	2 (1.6%)
Occupation	Farmer	100 (80.7%)
	Worker	7 (5.6%)
	Officer	4 (3.2%)
	Freelancer	2 (1.6%)
	Unemployment	1 (0.8%)
	Retired person	10 (8.1%)
Relationships between caregivers and dioxin victims	Wife	70 (56.4%)
	Parents	35 (28.2%)
	Siblings	8 (6.5%)
	Other relatives	11 (8.9%)
**Total**		**124 (100%)**

All caregivers were family members of victims. They were mainly victims’ wives (56.4%) or parents (28.2%) (Table 1).

### Information needs of caregivers in caring and rehabilitation for dioxin victims

The information needs of caregivers in caring and rehabilitation for dioxin victims were reported, including current sources of information, unmet information needs of caregivers in caring and rehabilitation for dioxin victims in regard to contents and forms. Altogether, 120 caregivers reported needing general information needs, accounting for 96.8% of respondents (Table 2).

**Table 2:** Information needs of caregivers in caring and rehabilitation for dioxin victims

Information needs of caregivers in caring and rehabilitation for dioxin victims (n = 124)	n	%
General information needs	120	96.8
No need for information	4	3.2

#### Current source of information about caring and rehabilitation for caregivers of dioxin victims

Caregivers identified several resources currently available for caregivers specific to dioxin. They reported that information from health staff counseling had the highest rate of providing sufficient information compared to other sources (32.2%). A sufficient level of accessing resources via family members or friends (9.7%), television (21.8%), online newspapers (11.3%), and radio (8.9%) were reported. The majority of respondents reported an acceptable level of accessing information about caring and rehabilitation via the radio (77.7%). In the study, 58.1% of caregivers reported not having information issues from the website due to unavailability. This is mainly due to the lack of access to the Internet and/or no information on dioxin made available to them (Table 3).

**Table 3:** Providing information about caring and rehabilitation for AO/dioxin caregivers

Providing information (%) (n = 124)	Sufficient	Acceptable	Few	Not available
Health staffs’ counselling	40 (32.2)	73 (58.9)	10 (8.1)	1 (0.8)
Family members or friends	12 (9.7)	60 (51.6)	41 (33.1)	7 (5.6)
Training courses	6 (4.9)	38 (30.6)	30 (24.2)	50 (40.3)
Leaflets or booklets	11 (8.9)	43 (34.7)	42 (33.9)	28 (22.5)
Radio	11 (8.9)	96 (77.4)	12 (9.7)	5 (4.0)
Television	27 (21.8)	86 (69.3)	8 (6.5)	3 (2.4)
Online newspapers	14 (11.3)	45 (36.3)	21 (16.9)	44 (35.5)
Social networks	9 (7.3)	42 (33.9)	25 (20.1z)	48 (38.7)
Website	4 (3.2)	29 (23.4)	19 (15.3)	72 (58.1)

Findings from qualitative interviews supported the quantitative findings above, demonstrating that the most common way of receiving information was from health staff, followed by television and radio. Leaflets or the Internet were much less likely to be available, thus not being accessed by most participants in the study.

There are different channels we can get access to, TV or radio from the commune, they talked about Agent Orange and support for dioxin victims. The commune health staff also came to provide counseling sometimes. I found no information on the Internet for dioxin victims (FGD 1, caregiver 3, female).

Caregivers who had a higher education level reported that they often chose official channels of communication to obtain information about dioxin-related policies and updates on programs for victims. In addition, reading digital or printed newspapers, or watching television was discussed as potential sources of information. However, it was reported by most of the participants that these resources do not have information on caring and rehabilitation.

I often read newspapers, watch TV, and watch VTV1 programs, or I read digital newspapers, but not much. The leaflets are not available at commune health stations on this issue (FGD 1, caregiver 4, female).

Caregivers reported that they were unable to access information related to supporting victims from the Internet, those who had attempted to obtain online intervention described it as very limited. Furthermore, the caregivers of victims are often elderly (71.8%), and there is a small number of them using the Internet or smartphones.

My wife used it only recently, we haven’t got that (Internet) before, for us, at this age, we do not use it much, that’s the first thing, the second thing is that it is not available in a lot of areas. Actually, I didn’t see anything about dioxin on the Internet (FGD 2, caregiver and victim 6, male).

Find out information about dioxin or rehabilitation for the victims... Yesterday, I opened but nothing was found (FGD 2, caregiver 7, female).

Besides the above sources of information, participants highlighted the need for regular training courses to disseminate knowledge on the practices of caring and rehabilitation as well as up-to-date information on victims. Dioxin caregivers want to train the victims at home and expressed their need for guidance materials in care and rehabilitation. Training programs were expressed by victims and caregivers in the study as an effective way of providing information for caregivers.

It is good to train the children because they are handicapped and the health professionals have those skills and it is easy for them to share with us. If the parents do not know, how can they help their children, how can they show the children (victims) do exercises correctly? That’s why as family members they need those materials. The materials are helpful for the health collaborators as well (FGD 2, caregiver and victim 6, male).

#### Needs for information contents

As reported in Table 4, the majority of these participants desired information about caring for dioxin victims (75.8%), psychological support (70.0%), and the harm of dioxin (60.8%). Information needs on support policies, nutrition, and rehabilitation practices at home were all high. The information needs from health staff and counselling on genetic/hereditary issues of dioxin victims were also high (54.2%).

**Table 4:** Details of information needs of caregivers in caring and rehabilitation for dioxin victims

Detailed information needs in caring and rehabilitation (n = 120)	n	%
Dioxin’s harms	80	66.7
Common types disability	40	33.3
Caring for AO/dioxin victims	91	75.8
Nutrition for AO/dioxin victims	77	64.2
Rehabilitation practice at home for AO/dioxin victims	66	55.0
How to make assistive and orthopedic devices	30	25.0
Policy information for AO/dioxin victims	73	60.8
Genetic counselling for AO/dioxin victims	65	54.2
Job counselling for AO/dioxin victims	34	28.3
Information about healthcare facilities for dioxin victims	65	54.2
Psychological support information	84	70.0
Ways to connect AO/dioxin victims’ association	46	38.3

The collected qualitative data supported quantitative findings and highlighted several issues including information on policies for victims, medical care and treatment, caring in daily lives, nutrition for victims, rehabilitation technique (rehabilitation exercises) training for patients and caregivers, psychological support, and financial support. Most of the focus group discussions and in-depth interviews indicated that dioxin caregivers were in urgent need of information.

*Needs for information on support policies for victims* Caregivers reported the need to get updated specific policies that support dioxin victims and caregivers. These policies should relate to financial support, or health insurance, social insurance support, or other government priorities that are available to them as victims of the war. Most victims and their families require support from the government as they are poor and many are incapable of having jobs. The following quote from a victim illustrates the limited financial support from the government.

I often tried to find out about the policies of the state regarding dioxin and updates of the support policies with the hope that we can get something. But the information is very scarce (IDI 3, victim, male).

At the moment, my support level as F1 generation (of dioxin victim) is 974.000 VND per month. My dad was verified and recognized as a dioxin victim and I was verified as a person who suffered from the toxic chemical. The F2 generation people can get 540.000 VND per month. I don’t have any other support (IDI 4, victim, female).

The health insurance policy supports dioxin victims with 100% health insurance coverage. Health insurance could cover the payment in case the patient has a disability verification certificate. As reported, they used health insurance for medical care purposes.

Regarding health insurance, I had 100% prevention of sickness coverage, but I only used it for common illnesses, aching in the limps so I get some medicines home to take. (IDI 12, victim, male).

In some cases, the poor families could get subsidy support for public schooling.

My family was recognized as a poor household, so my daughter can have some reduction in tuition fees at the school (IDI 9, caregiver, female).

*Needs for information on medical care and psychological support* Victims and their caregivers frequently reported a need for medical support and services, apart from information regarding health insurance as mentioned above. Caregivers reported that dioxin patients need regular health check-ups and better treatment with medications. Some caregivers in the study shared that families affected by dioxin required psychological support. Many participants are aware that dioxin affects the health of victims and creates mutations in their genes. Most families and dioxin victims reported having constant concerns about the future of their next generations particularly if they get married. The psychological burden is obvious among caregivers in this study. Commonly caregivers reported that victims find it difficult to cope with daily life, particularly concerning mood or anxiety. Caregivers reported a need for psychological services to assist patients to manage these challenges.

... diagnosing diseases and all that, we need to rely on the health sector and then we need projects that have regular health checks at communes for the victims, first is counselling, and second is providing partial medical support for us (FGD 2, caregiver 8, female).

… About spiritual support, we also need, but indeed, my children have been sick since childhood. We still live with our children and grandchildren; we don’t need someone to come to our home for psychological support. However, building groups that we can share with each other is good. We are also in the Orange Agent Association (FGD 2, caregiver and victim 6, male).

I don’t know if my son gets married, his children will be normal or not. He has the first child, and then the second child with disabilities, I still hope the third child will be normal, not like the first two. I am worried all the time (IDI 10, victim, male).

*Needs for information on daily care and rehabilitation at home* The need for information on daily care, including basic care, nutrition, and exercises was commonly mentioned during interviews with caregivers. Most caregivers did not have much guidance or were trained to care for victims by health professionals such as daily care or providing psychological support for victims. Some elderly victims who are more than 70 years old shared that they find it a heavy burden because they have to look after the other victims all the time who are their younger relatives.

Yes, it is great if the caregivers and victims know about what food they should eat, how to care for them, which exercises they should follow, and medications as well (IDI 2, victim, male).

I find it difficult to care for my grandson, he’s running around all the time, and sometimes he ran out of the house, and I could not follow him. My life was so miserable you know [cry] (FGD 2, caregiver and victim 9, male).

Caregivers expressed their desire to get information and have training regarding rehabilitation so that they can guide their family members who are victims to do rehabilitation exercises at home daily. Caregivers reported that this information should be delivered by rehabilitation specialists or trained health professionals. Due to the complex needs of victims reported by caregivers, they were concerned that they do not provide the correct level of care for them. Providing hands-on practices is considered the most effective way of transferring knowledge and skills to victims and their caregivers. This idea was strongly supported by caregivers, victims, and health staff who participated in the qualitative interviews. As one caregiver in a focus group discussion illustrates:

I think I need the knowledge of rehabilitation to do for the victims... In general, it’s great if someone could come to show/teach us how to guide my son to do rehabilitation exercises at home. We don’t have any machines to help so it’s better if we can do exercises by ourselves (FGD 2, caregiver 7, female).

Apart from the need for health staff to provide rehabilitation training for caregivers and victims, some participants mentioned the need for having assistive devices at home to alleviate the caring burden for caregivers at homes, such as wheelchairs, or crutches:

My opinion is that I wish to have the equipment to rehabilitate for families’ members. It’s the best if the government or hospitals periodically provides training for people who are directly involved in caring for the victim (FGD 1, caregiver and victim 5, female).

It is very important to have periodic training classes on how to provide home care, and rehabilitation at home for victims (FGD 2, caregiver and victim 9, male).

*Needs for information on financial support for victims and caregivers* Caregivers commonly identified the need for financial support as many families affected by dioxin are very poor and cannot work (due to comorbidities). In the context where both caregiver and victim are not able to earn their living, the financial support policy is of significant importance. Caregivers suggested that financial support could be in the form of providing more subsidies for health insurance or providing higher monthly allowances.

We need the government support in finance so that we can afford to go to hospitals. Agent Orange victims need a lot more financial support. (IDI 13, victim, male).

However, one participant shared that he had to refuse the government support for his daughter who is a dioxin victim (second generation) due to the concern that it will affect his daughter’s marriage in the future. It is a common belief that it is more difficult for girls than boys to get married if they have health conditions that are related to hereditary problems, and dioxin will make the situation worse for girls.

Both my son and my daughter can get the allowance from the government for dioxin-affected people, but I didn’t get it for my daughter because I am concerned that my daughter cannot get married if people know she is a dioxin victim. I only get it for my son (IDI 14, caregiver and victim, male).

##### Needs for information from different channels and forms

As reported in the survey findings, 120 out of 124 (96.8%) caregivers reported that they had general information needs with various information channels. The study investigated the three most favorite forms of information that those dioxin caregivers desired. The most chosen information form was counseling by health staff (85.0%). The percentages of caregivers interested in receiving information via television and radio channels were 75.0% and 65.8%, respectively (Table 5).

**Table 5:** Demand for information forms of caregivers in caring and rehabilitation for dioxin victims

Demand for information forms (n = 120)	n	%
Medical staffs’ counselling	102	85.0
Family members, friends	39	32.5
Training courses	38	31.7
Printed materials	48	40.0
Radio	79	65.8
Television	90	75.0
Internet newspapers	9	7.5
Social networks	11	9.2
Website	7	5.8

In this study, participants were in favor of having advice or counselling from health professionals followed by TV and community loudspeakers. These findings were consistent with the findings mentioned earlier on the current situation of information access (Table 2) where counselling from health staff, TV, and loudspeakers are reportedly the most accessible channels. Caregivers often desired information from rich-information and accessible sources, so the need to provide this along with advice from health workers is considered appropriate. This is the most desired communication as shared by several caregivers participating in this study.

I think I need the knowledge of rehabilitation for the victims... In general, regarding rehabilitation, if there are health professionals coming our houses to show rehabilitation exercises, they will recover. We want to have machines to do exercise at home, but we could not afford them (FGD 1, caregiver 1, female).

The needs for other printed materials such as books, booklets, newspapers, and leaflets were also mentioned as a channel of information to which participants want to get access. However, these methods are not all available or accessible to victims and caregivers in many cases.

The printed documents can be sent to the communes for caregivers for further information, but now we all don’t have that kind of materials at localities (FGD 2, caregiver 10, male).

When we investigated the need for building a supportive website, 48/124 (38.7%) dioxin caregivers reported that they found it was necessary or very necessary to have a website with information and skills for caring and rehabilitation.

Information accessed from the Internet via smart TV, computer, or smartphones was reported in the qualitative data. However, this channel was not common among caregivers in the study. In explanation of this situation, two reasons were given: first, participants did not use the Internet frequently as the majority of them were elderly; and second, the information regarding care and rehabilitation on the Internet was still very limited. Some participants highlighted the advantages of using the Internet to get information and improve caring skills for caregivers.

My house has Internet, I can access through TV, but most of the time I actually could not find any information I need on dioxin or for dioxin victims on TV or on the Internet (FGD 2, caregiver 10, male).

We can use the website to get information about dioxin for better prevention of sickness and to share information and caring skills with others in the same situation, neighbours, and community (IDI 2, victim, male).

It is understandable that the information on the Internet should be visualized with pictures, videos and should be accessible at any time.

The information on the website should have pictures so that we can easily understand it. If we could hear and see regularly, we won’t forget. The pictures should be arranged in a way that it’s easy to understand for normal people, if it’s complicated it’s only appropriate for like higher education people, we will not be able to understand (IDI 2, victim, female).

## Discussions

This is the first study to investigate the information needs of caregivers of victims affected by dioxin in Thai Binh province, Vietnam. The findings highlight the absence of supportive information and the urgent requirement for the development of supportive systems. The information needs in this study, however, does not include information needs to take care for themselves as caregivers.

### General characteristics of caregivers of dioxin victims

Findings of this study identify the caregivers of dioxin victims are more likely to be elderly female family members, particularly wives of the victims (Table 1). This result is consistent with the predominating rate of female informal caregivers of cancer patients in Vietnam [14]. However, this finding is not consistent with other Vietnam caregiver studies of disabilities, which identify these caregivers are more likely parents [22]. A possible explanation is that dioxin victims are more likely to be adult males who were exposed to dioxin during the Vietnam War from 1961 to 1972. Therefore, caregivers are often older wives of these dioxin victims. This finding is of importance for the development of information and support that should be targeted toward an older population.

This finding about the characteristic of participants in this study is different from a study by Riewpaiboon and colleagues on caregivers for people with disabilities in Vietnam [22]. In their study, caregivers were parents and accounted for 49% of participants, while caregivers who were the spouses represented only 34%. A potential reason for this difference is that Riewpaiboon’s study participants were caregivers for people with all different types of disabilities, such as mobility disability, mental or intellectual impairments and both mobility and mental impairments [22]. On the other hand, participants in our current study are mostly male victims’ caregivers. These victims were exposed directly to dioxin in the Vietnam War. Meanwhile, participants who were parents of dioxin victims only accounted for 28.2%. It is because the inclusion criteria for dioxin victims also included the second and third generations of the victims directly exposed to dioxin during the Vietnam War. Therefore, providing information support should prioritize female elderly (of the first generation) and parents (of the second generation) of victims.

In addition to caregivers being older and female, many caregivers report living with a low income (98.4%) and low educational level (68.6%). The characteristics may represent the discrimination of females in the rural area. They are often excluded from formal education, complete unpaid activities, such as being a housewife thus have a high rate of unemployment [28].

### Needs for information on care and rehabilitation of dioxin victims

This study identifies that the information needs of dioxin caregivers is high with 96.8% sharing that they need to be provided care and rehabilitation. Caregivers for dioxin victims have crucial roles in care and support for victims. This result is similar to the research on the need of providing information for caregivers of cancer patients in Vietnam, showing that caregivers have multiple roles in supporting the patients, making decisions related to care and treatment for patients. Their burden of care required information related to care, treatment assistance, medical and social services for patients [23].

This current study demonstrated clear evidence of the information needs of caregivers for dioxin victims in Vietnam from qualitative interviews, which is consitent through quantitative results. However, most caregivers in the study agreed that the available information is very limited. A previous study also highlighted the need to provide hands-on, effective skills training for caregivers including those who provide care for dioxin patients [29].

## Implications for practice and policy

### Contents of information needed

*Needs for information on daily care, psychological support and rehabilitation* The contents of information that caregivers need to know are how to provide daily care for the victim, financial support (monthly allowance and health insurance subsidy), information on support policy, nutrition, harms of dioxin, and rehabilitation exercises to guide victims at home for the victims. Caregivers who are family members in Vietnam have almost no support services, nor are they trained in care and rehabilitation for victims. Some previous community-based rehabilitation activities on disability, victims and their family caregivers were conducted in rural areas in Vietnam, but the results were very modest. Most participants reported that these activities were too theoretical, and transferring practical knowledge and skills were very limited [29].

The high need for information regarding nutrition was notable. This finding is similar to caregivers’ chronic health conditions [13, 30]. Moreover, some studies have shown that the importance of understanding of nutrition for citizens living in dioxin contaminated environments. Advices on nutrition may be benificial for caregivers, victims, and locals [31, 32].

Needs for information on how to provide psychological support for caregivers and victims such as sharing stories from other caregivers and families, coping with worries and uncertainty about the future of the next generations, and hereditary issues are also unmet. These findings are consitent with studies on caregiver of cancer patients. Cancer caregivers are more likely to seek information to be able to support patients psychologically [30]. Additionally, dioxin victims and their caregivers often deal with stigmatization. The stigmatization can be a barrier for people with disability and their families to well-being and inclusion, especilly in dioxin contamination areas [33]. Therefore, the information about how to decrease the stigmatization and coping skills may be effective support for dioxin victims’s caregivers. Policies focusing on financial support and stigma prevention should be also considered to support family caregivers and dioxin victims by stakeholders.

*Needs for information on financial policy support and health insurance support* It is very common in the interviews that participants want to gain more updated information about the support policies for dioxin victims. Financial support or subsidization of health insurance are often shared in the interviews. Even though victims can have a small monthly allowance, it could considerably alleviate the burden on their families.

The current policy in Vietnam stipulated that only the victims and the second generation (children of people directly exposed to dioxin) can get benefits from the government policies [34]. Veterans who have a 61% reduction in working ability (moderate and severe level) can get health insurance for free. In Vietnam, caregivers of dioxin victims have no support from the government, except for those caring for people with very severe disabilities and completely depend on caregivers - they can have a small monthly allowance.

### The channels of information needed

Regarding the information channels, the study highlighted the importance of providing information through health care professionals, TV, radio and expanding the opportunities to have digital support for caregivers and their victims. Most participants wanted information on care and rehabilitation for victims from counseling by health workers (85.0%). This result is similar to findings of a previous study that highlighted that caregivers preferred to be informed by and to discuss with health care professionals [35]. Caregivers want health professional support not only for information but also for receiving other support from medical staff so that they could understand the information they receive properly. Counseling from health workers is preferred by dioxin victims because they can interact with and have explanations from health care professionals. The study showed the important roles and necessity of providing training for health workers at the grassroots level as they can provide counseling and training for caregivers in the community on care and rehabilitation. Besides, choosing radio or TV can be feasible for delivering the information to dioxin caregivers based on currently available channels of information and caregivers’ preferences. Also, incorporation of information from medical staff through TV or radio channels may need to consider to apply.

In addition, dioxin caregivers had a high need for hands-on knowledge and skills regarding the care and rehabilitation of victims. Therefore, an empowerment approach can be suitable for dioxin caregivers. This finding fits with the goals and action program of community-based rehabilitation. In this program, core human resource is based on community force, such as families, and people with disability [25].

The study suggested that an online website for caregivers and victims should be considered. Although it might not be appropriate for most caregivers in this study (most of them are elderly and might not be familiar with finding information on the Internet), providing online support could be effective for the second or third generations of victims. In particular, during COVID-19 pandemic time, digital support has been promoted. Previous studies showed the effectiveness of online support for patients and caregivers as knowledge and skills training could be made available via the Internet. In addition, the rate of people using the Internet in Vietnam is increasing and has been noted to be as high as 70% [36], which can facilitate to implementation of an online support system for caregivers. Despite the significant Internet coverage, enhancing the Internet accessibility for the caregiver in rural areas may be necessary before implementing any online support. Besides, sharing from peers, or people with the similar situation could be a helpful and informative coping strategy, thus providing psychological support for caregivers [15]. This study only focused on the needs of caregivers regarding care and rehabilitation for dioxin victims, further studies are needed to have a better understanding of the personal needs of these caregivers.

## Conclusion

This study showed the high need for information for caregivers of dioxin victims across different generations. The information may help caregivers (who experience a significant burden in caring for the victims) with knowledge and skills for providing better daily care and rehabilitation for victims and themselves, as a large number of caregivers are also dioxin victims. Greater information on the support policies (i.e. financial support in monthly allowance or health insurance support) for dioxin victims especially those who have severe disabilities and are unable to work is highly needed. Training for health professionals at the grassroots level who in turn will train caregivers in communities should be promoted, as well as apart from other common modes of information delivery like TV, radio, and leaflets. Findings suggest the potential of developing an online support system for caregivers, victims and health staff that provides knowledge, skills training with videos, pictures, and story sharing.

## Data Availability

The datasets generated and/or analyzed during the current study are not publicly available due to the privacy of research participants but are available from the corresponding author on reasonable request.

## Declarations

**Ethical approval and consent to participate**

The study received ethics approval from the Hanoi University Institutional Review Board (Approval number 489/2019/YTCC-HD). All primary data collection was conducted after obtaining informed consent from each participant and ensuring the reporting of results protected the participants’ identities. In this study, all methods were carried out in accordance with relevant guidelines and regulations.

## Abbreviations

AO: Agent Orange
IDI: In-depth Interview
FGD: Focus Group Discussion
LMICs: low-and-middle-income countries
